# Ergotine as a Hæmostatic

**Published:** 1872-01

**Authors:** 


Ergotine as a Hæmostatic.—The *Bulletin General Therapeutique* quotes from the British Medical Journal a case of Dr. Jamieson, of Berwick, in which the sub-cutaneous injection of ergotine proved successful in arresting pulmory hemorrhage on three separate occasions in a man aged forty-one. The *Bulletin General*, following Dr. Jamieson, ascribes the first idea of this excellent mode of employing ergotine as a hæmostatic to Dr. G. W. Balfour. The proposal, however, came originally from Langenbeck, of Berlin. Whenever the hemorrhage is of snch a nature that it can be arrested by simple contraction of the smaller arteries, good ergotine is, when thus employed, sure to be successful; and as the sub-cutaneous injection of the arm, or some fleshy part, is productive of not the slightest inconvenience, it is always worthy of trial. Three to six grains are sufficient for one injection, and it may be repeated in three minutes, if necessary. The action is almost instantaneous. A prepared solution may be kept in readiness, with a small proportion of spirit of glycerine to preserve it.

